# Integrated Analysis of Metabolomics and Lipidomics in Plasma of T2DM Patients with Diabetic Retinopathy

**DOI:** 10.3390/pharmaceutics14122751

**Published:** 2022-12-08

**Authors:** Chun Ding, Nan Wang, Zicong Wang, Wenyun Yue, Bingyan Li, Jun Zeng, Shigeo Yoshida, Yan Yang, Yedi Zhou

**Affiliations:** 1 Department of Ophthalmology, The Second Xiangya Hospital of Central South University, Changsha 410011, China; 2 Hunan Clinical Research Center of Ophthalmic Disease, The Second Xiangya Hospital of Central South University, Changsha 410011, China; 3 National Clinical Research Center for Metabolic Diseases, The Second Xiangya Hospital of Central South University, Changsha 410011, China; 4 Department of Ophthalmology, Kurume University School of Medicine, Kurume 830-0011, Japan

**Keywords:** metabolomics, lipidomics, biomarker, diagnosis, diabetic retinopathy, proliferative diabetic retinopathy, non-proliferative diabetic retinopathy

## Abstract

Diabetic retinopathy (DR) is a major cause of blindness worldwide and may be non-proliferative (NPDR) or proliferative (PDR). To Investig.gate the metabolomic and lipidomic characteristics of plasma in DR patients, plasma samples were collected from patients with type 2 diabetes mellitus (DR group) with PDR (*n* = 27), NPDR (*n* = 18), or no retinopathy (controls, *n* = 21). Levels of 54 and 41 metabolites were significantly altered in the plasma of DR patients under positive and negative ion modes, respectively. By subgroup analysis, 74 and 29 significantly changed plasma metabolites were detected in PDR patients compared with NPDR patients under positive and negative ion modes, respectively. KEGG analysis indicated that pathways such as biosynthesis of amino acids and neuroactive ligand-receptor interaction were among the most enriched pathways in altered metabolites in the DR group and PDR subgroup. Moreover, a total of 26 and 41 lipids were significantly changed in the DR group and the PDR subgroup, respectively. The panel using the 29-item index could discriminate effectively between diabetic patients with and without retinopathy, and the panel of 22 items showed effective discrimination between PDR and NPDR. These results provide a basis for further research into the therapeutic targets associated with these metabolite and lipid alterations.

## 1. Introduction

Diabetic retinopathy (DR), one of the most common diabetic microangiopathies, is a leading cause of vision loss in adults [1]. In recent years, the number of diabetes patients has risen sharply globally, reaching nearly 120 million in China (the highest number per country), where it is expected to exceed 140 million by 2030 [2]. In patients with type 2 diabetes mellitus (T2DM), the overall prevalence of DR exceeds 30% [3]. The pathological progression of DR occurs in two stages: non-proliferative DR (NPDR) and proliferative DR (PDR) [4]. Early diagnosis and treatment of DR are important to prevent the progression of vision loss and blindness.

In clinical practice, DR is mainly screened by fundus examination combined with ophthalmic imaging such as fluorescein angiography [5]. However, many patients have ocular comorbidities (such as cataracts [6]), which impede observation and diagnosis. Therefore, exploring potential biomarkers with high sensitivity and specificity is of great clinical significance for early diagnosis, treatment, monitoring, and accurate prognosis of DR. Recently, the roles of many key regulatory factors in the development and progression of DR, such as vascular endothelial growth factor [7], platelet-derived growth factor [8], and periostin [9], have been revealed, but the mechanism underpinning DR pathogenesis remains unclear.

Metabolomics is a novel high-throughput profiling technique to comprehensively reveal the levels of small-molecule metabolites of size < 1500 Da [10]. This approach provides indicators of health status and has been widely applied in studies of many diseases, such as cancers [11,12,13], diabetes [14], neurological disorders [15,16,17], and others. Metabolomics has also been used in ophthalmological research on retinopathy of prematurity (ROP) [18], myopia [19], polypoidal choroidal vasculopathy, and age-related macular degeneration [20]. Wang et al. [21] demonstrated notable changes in metabolomics profiling and metabolic pathways in plasma and vitreous of patients with PDR. Therefore, metabolomics may prove a useful tool to identify early biomarkers of retinal and choroidal diseases [22].

Lipidomics is a more targeted model used to systemically analyze the composition and expression levels of lipid molecules [23] and has also been applied in research on diabetes [24,25] and DR [26,27,28]. Recently, an increasing number of studies have used both metabolomics and lipidomics to Investig.gate potential biomarkers and mechanisms [29,30] and have expanded the understanding of the pathogenesis of many diseases.

In the current study, plasma samples were collected from 66 T2DM patients aged 42–79 years, including 45 patients with and 21 without retinopathy. Untargeted metabolomics and lipidomics were comprehensively analyzed using these plasma samples. Bioinformatics analysis was then performed to predict possible relevant pathways. A random forest approach was used to assess the clinical values of the panel according to the integrated metabolomics and lipidomics analyses for the diagnosis of DR.

## 2. Materials and Methods

### 2.1. Study Subjects and Sample Collection

A total of 66 T2DM patients recruited from the Second Xiangya Hospital of Central South University were included in this study. The included patients were diagnosed with T2DM and aged over 40 years old. 

PDR was diagnosed by recognizing retinal neovascular tufts, vitreous hemorrhage, and fibrovascular membranes, the diagnosis of PDR and NPDR was determined by fluorescein fundus angiography or fundus examination, and the exclusion criteria were as follows: (1) combined with the following ocular conditions: neovascular glaucoma or rhegmatogenous retinal detachment caused by PDR and other severe retinal/choroidal diseases; (2) previous ocular surgery or therapeutic treatment histories such as vitrectomy, laser photocoagulation, or intravitreal injection; (3) combined with severe systemic diseases and coagulation abnormalities. The study protocol was approved by the Ethics Committee of the Second Xiangya Hospital of Central South University (number: 2021LY037) and conformed to the principles of the Declaration of Helsinki. Written informed consent has been signed by the patients.

A whole blood sample (2–3 mL) was collected from each included patient in a fasting state, using a heparin anticoagulant collection tube. Samples were centrifuged for 10 min (2000× *g*, 4 °C), and the supernatant (plasma) was immediately frozen in liquid nitrogen for metabolomic and lipidomic analyses after storage at −80 °C.

### 2.2. Sample Preparation

Plasma samples (100 μL) were thawed and incubated in cold methanol/acetonitrile (1:1, *v*/*v*, 400 μL) mix, and centrifuged for 15 min (14,000× *g*, 4 °C) for metabolomic analysis. The supernatant was dried in a vacuum centrifuge, then treated with 100 μL acetonitrile/water (1:1, *v/v*) solvent to redissolve the sample.

For lipidomic analysis, samples were incubated with water (200 μL). The mixture was vortexed for 5 s, and pre-cooled methanol (240 μL) was vortexed for 30 s. The sample was added with MTBE (800 μL) and sonicated for 20 min at 4 °C, and allowed to stand for 30 min at room temperature. The solution was centrifuged for 15 min (14,000× *g*, 4 °C), and the upper organic solvent layer was obtained and dried in nitrogen.

### 2.3. Metabolite Measurements

Using UHPLC (1290 infinity LC, Agilent Technologies, Santa Clara, CA, USA) and a quadrupole time-of-flight mass spectrometer (triplet of 6600; AB Sciex), measurements were made as described previously [18] with some modifications. HILIC separations with 2.1 mm × 100 mm ACQUIY UPLC BEH 1.7 µm columns (Waters, Ireland) were used for analysis. The mobile phase contained A = 25 mM ammonium acetate and 25 mM ammonium hydroxide in water and B = acetonitrile in both ESI positive and negative modes. The gradient was 95% B for 0.5 min, linearly decreased to 65% over 6.5 min, decreased to 40% over 1 min, held for 1 min, increased to 95% over 0.1 min, and the re-equilibration period was 3 min.

### 2.4. Lipid Measurements

Lipid analysis was performed using LC-MS/MS. After reversed-phase chromatography, a CSH C18 column (1.7 μm, 2.1 mm × 100 mm, Waters) was used for LC separation. Lipid extracts were reconstituted with 200 µL 90% isopropanol/acetonitrile, centrifuged at 14,000 g for 15 min, and 3 µL samples were finally injected. Solvent A was acetonitrile-water (6:4, *v*/*v*), 0.1% formic acid, and 0.1 Mm ammonium formate; Solvent B was acetonitrile–isopropanol (1:9, *v*/*v*) with 0.1% formic acid and 0.1 Mm ammonium formate. The initial mobile phase was 30% solvent B and the flow rate was 300 μL/min for 2 min, followed by a linear increase to 100% solvent B over 23 min, and then equilibration at 5% solvent B for 10 min.

Mass spectra were acquired using Q-Exactive Plus in positive and negative mode, respectively. All measured ESI parameters were optimized and preset: source temperature, 300 °C, capillary temperature 350 °C, ion spray voltage 3000 V, s-lens RF level 50%, and instrument scan range M/Z 200–1800. 

Lipid Search is a lipid species identification search engine based on MS/MS mathematics with more than 30 lipids and more than 1,500,000 fragment ions in its database. Mass tolerances were set to 5 ppm for both precursors and fragments.

### 2.5. Bioinformatics Analysis

The Kyoto Encyclopedia of Genes and Genomes (KEGG) database (https://www.kegg.jp/, accessed on 1 April 2022) was used for KEGG pathway analysis to identify the potential signaling pathways involved in changes to metabolic features. The random forest process was implemented in R using the rfPermute and randomForest packages (version 4.2.1). Receiver operating characteristic (ROC) curves were employed for prediction.

### 2.6. Statistical Analysis 

For calculating the demographic characteristics of the participants, comparisons of numerical variables between the three groups were made using one-way ANOVA, and comparisons of categorical variables were made using the Chi-square test. Significantly changed metabolites and lipids by metabolomics and lipidomics analyses were screened using the variable importance in projection (VIP) > 1 obtained from an orthogonal partial least squares discriminant analysis (OPLS-DA) model and student’s *t*-test *p*-values (*p* < 0.05). Analysis of covariance was applied to examine the effect of sex difference on differential metabolites and lipids. The significance of correlations was determined by threshold values of |R| > 0.5 and *p* < 0.05.

## 3. Results

### 3.1. Demographic Characteristics of the Study Participants

As shown in Table 1, a total of 66 patients with T2DM were included in the study, including 27 with PDR (female: 48.15%), 18 with NPDR (female: 61.11%), and 21 without retinopathy as controls (female: 42.86%). No significant difference between groups was found in gender, age, BMI, fasting blood glucose, and HbA1c. A significant difference was found between groups in the duration of diabetic disease (*p* = 0.0151), being 13.00 ± 6.50 years in the PDR group, 11.00 ± 4.75 in NPDR, and 7.91 ± 5.80 in controls.

### 3.2. Metabolomic Analysis of Participants’ Plasma

**Figure 1:**
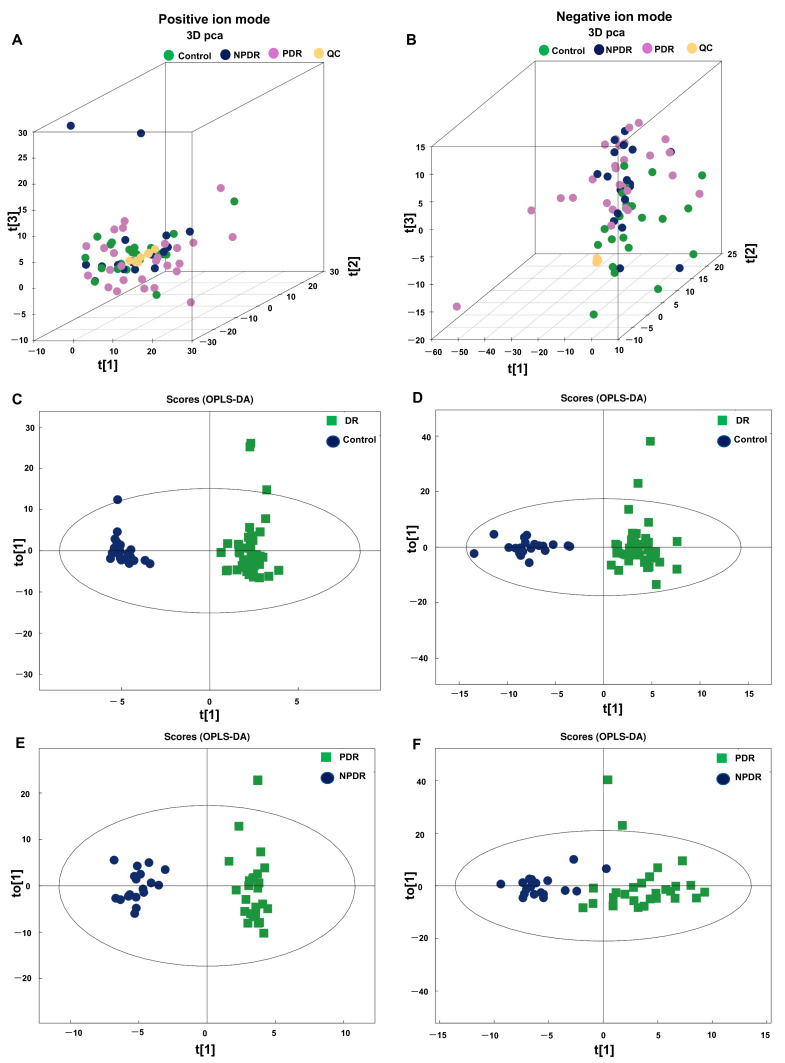
Multivariate statistical analysis of the metabolomics analyses. 3D PCA plots of the plasma metabolomics data of the included plasma samples under positive (**A**) and negative ion mode (**B**). OPLS-DA with score plot of DR group and control group under positive (**C**) and negative ion mode (**D**). OPLS-DA with a score plot of PDR subgroup and NPDR subgroup under the positive (**E**) and negative ion mode (**F**). PCA, principal component analysis; DR, diabetic retinopathy; OPLS-DA, orthogonal partial least squares discriminant analysis; PDR, proliferative diabetic retinopathy; NPDR, non-proliferative diabetic retinopathy.

To uncover the metabolites that changed in DR, untargeted metabolomics analysis was performed on plasma samples from all participants. As can be seen in the principal component analysis (PCA) plots, the QC samples were relatively acquired in both positive and negative ion modes (Figure 1A,B, respectively). Results of OPLS-DA showed distinction in both positive and negative ion modes between the DR and control groups (Figure 1C,D) and between PDR and NPDR subgroups (Figure 1E,F).

**Figure 2:**
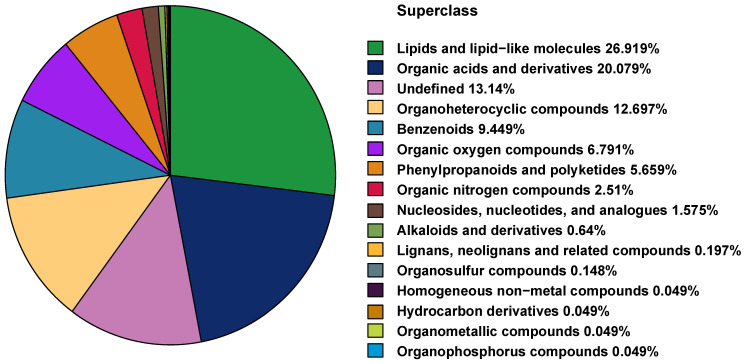
Classification of detected metabolites in plasma samples according to their chemical taxonomy.

A total of 1500 and 532 metabolites were detected in both positive and negative ion modes. We classified the identified metabolites according to the chemical classification shown in the pie chart (Figure 2), the top three superclasses being: lipids and lipid-like molecules, organic acids and their derivatives, and organic heterocycles.

**Figure 3:**
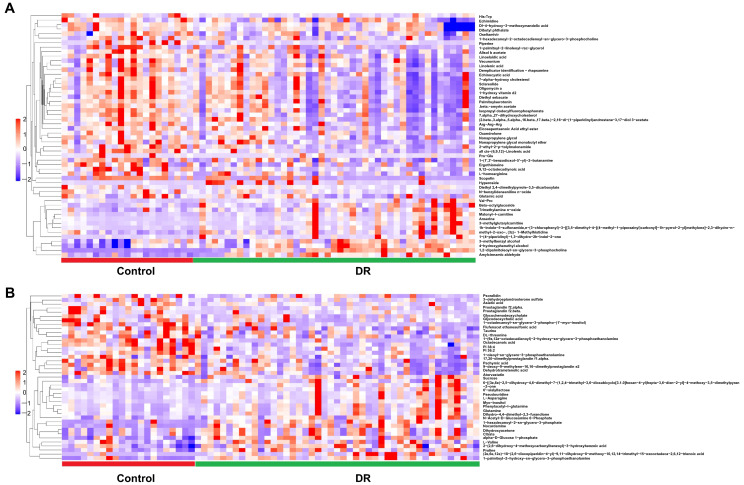
Hierarchical clustering of significantly changed metabolites in DR samples compared with controls under the positive (**A**) and negative ion modes (**B**). Blue indicates a low level, red indicates a high level of each metabolite, and similarly, hereinafter.

**Figure 4:**
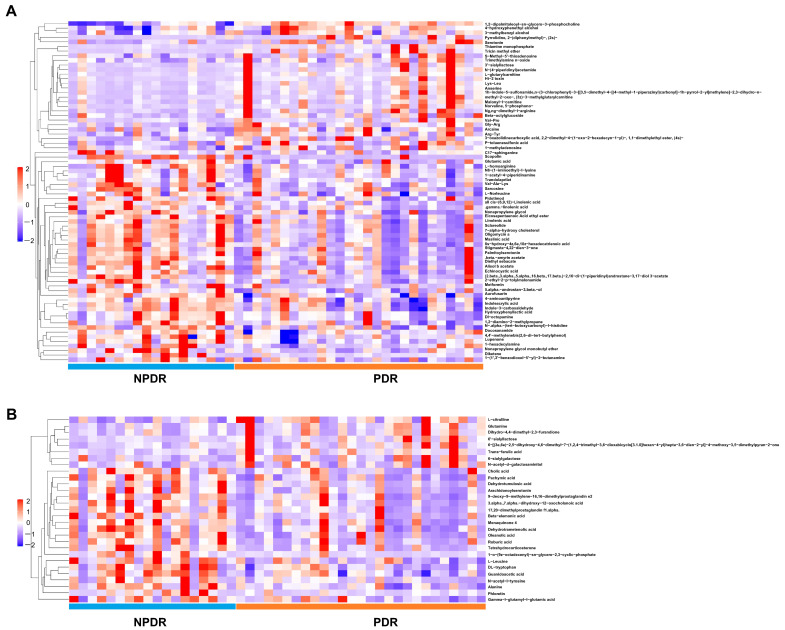
Hierarchical clustering of significantly changed metabolites in PDR samples compared with NPDR samples under the positive (**A**) and negative ion modes (**B**).

A total of 54 and 41 metabolites were significantly differently altered between DR and control groups under positive (Figure 3A and Supplementary Table S1) and negative ion modes (Figure 3B and Supplementary Table S2), respectively. In addition, 74 and 29 metabolites were differently altered significantly between PDR and NPDR subgroups under positive (Figure 4A and Supplementary Table S3) and negative ion modes, respectively (Figure 4B and Supplementary Table S4).

As shown by the Venn diagram in Supplementary Figure S1A,B, intersections were found between significantly altered metabolites in PDR, NPDR, and control groups. In the positive ion mode, the levels of 4-hydroxyphenethyl alcohol, and 1,2-dipalmitoleoyl-sn-glycero-3-phosphocholine were significantly altered in each comparison and in the negative ion mode, the levels of pachymic acid, and dehydrotrametenolic acid were significantly altered in each comparison.

### 3.3. KEGG Pathway Analysis of Altered Metabolites

**Figure 5:**
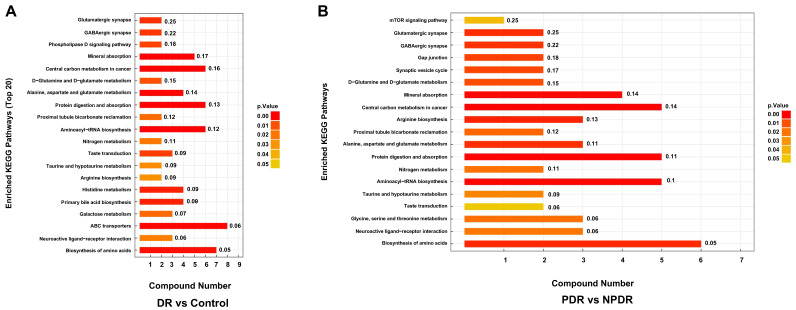
Enriched pathways of metabolites with significantly different levels in the DR group compared with controls (**A**), and PDR subgroup compared with the NPDR subgroup (**B**). KEGG, Kyoto Encyclopedia of Genes and Genomes.

As shown in Figure 5A, enriched pathways associated with DR included glutamatergic synapse, GABAergic synapse, phospholipase D signaling pathway, mineral absorption, biosynthesis of amino acids, and others. Pathways enriched differentially between PDR and NPDR included the mTOR signaling pathway, glutamatergic synapse, GABAergic synapse, gap junction, and biosynthesis of amino acids (Figure 5B).

### 3.4. Lipidomics Profiling of Plasma from the Participants

**Figure 6:**
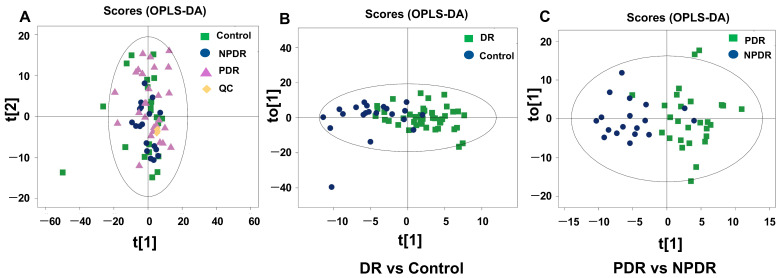
Multivariate statistical analysis of the lipidomics analyses. PCA plot of the plasma lipidomics data of the included plasma samples (**A**). OPLS-DA with score plot of DR group and control group (**B**). OPLS-DA with score plot of PDR subgroup and NPDR subgroup (**C**).

As shown in Figure 2, the “lipid class and lipid-like molecule” superclass was the major class of metabolites identified. Figure 6A shows that the QC samples were clustered in the PCA plot of lipidomics. The OPLS-DA plots indicated separations between the DR and control groups (Figure 6B), and the PDR and NPDR subgroups (Figure 6C), but these separations are not as well as the metabolomics analyses.

Next, we assessed the total level of lipids in different samples. No significant differences were found between the DR and control groups (Supplementary Figure S2A) or between the PDR and NPDR subgroups (Supplementary Figure S2B).

**Figure 7:**
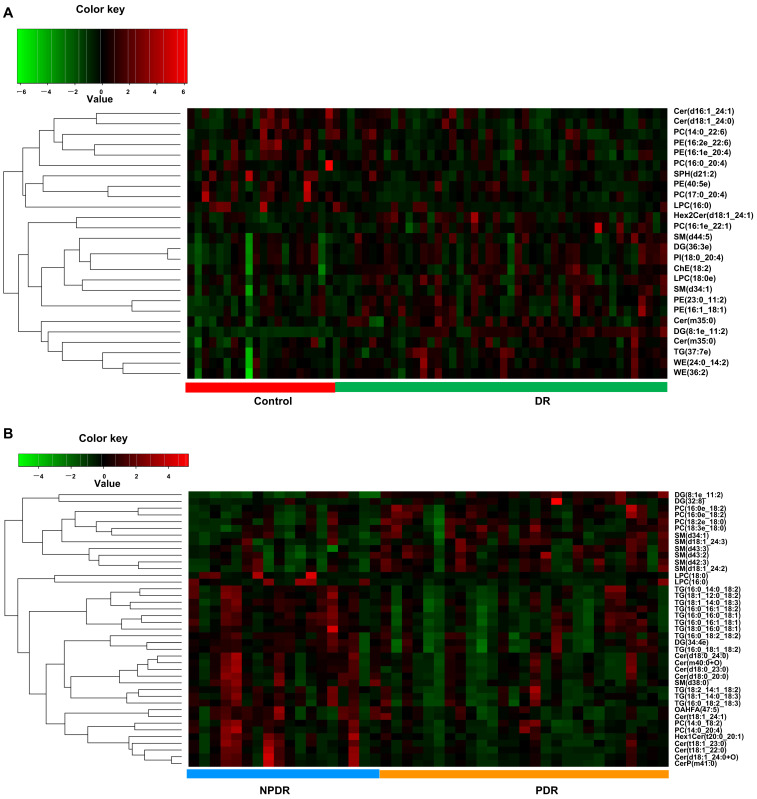
Hierarchical clustering of significantly changed lipids in DR samples compared with controls (**A**), and in PDR samples compared with NPDR samples (**B**). Green indicates a low level, red indicates a high level of each metabolite.

Twenty-six lipids were significantly differently altered in the DR group compared with the control group (Figure 7A and Supplementary Table S5), while 41 lipids were significantly differently altered in the PDR subgroup compared with the NPDR subgroup (Figure 7B and Supplementary Table S6).

Intersections between the significantly altered lipids in PDR, NPDR, and control groups showed that the level of DG(8:1e_11:2)+NH4 was significantly altered in each comparison (Supplementary Figure S3).

**Figure 8:**
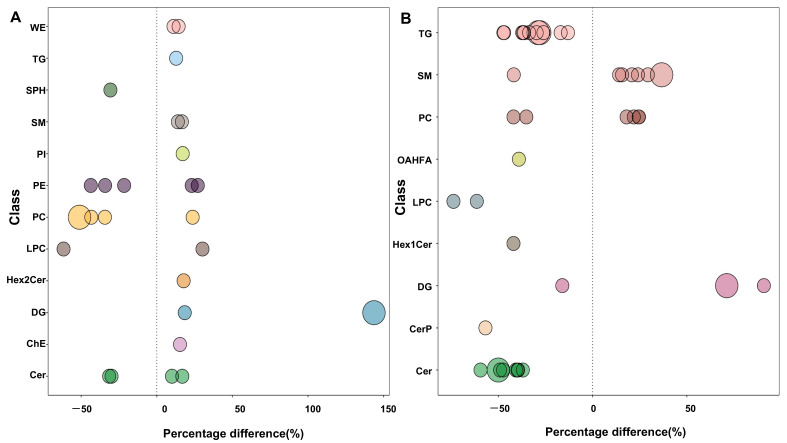
Bubble plots of classification of significantly altered lipid molecules in the DR vs. control groups (**A**), and PDR vs. NPDR subgroups (**B**). The bubble size indicates the significance of the differences (small size: 0.01 < *p* < 0.05; large size: *p* < 0.01). WE, wax exters; TG, triglyceride; SPH, sphingosine; SM, sphingomyelin; PI, phosphatidylinositol; PE, phosphatidylethanolamine; PC, phosphatidylcholine; LPC, lysophosphatidylcholine; Hex2Cer, hexosyl ceramide; DG, diglyceride; ChE, cholesterol ester; Cer, ceramides; OAHFA, (O-acyl)-1-hydroxy fatty acid; CerP, ceramides phosphate.

Bubble plots (Figure 8) show the significance of the differences and the classification of different lipids. As shown in Figure 8A, phosphatidylethanolamine (PE), phosphatidylcholine (PC), and ceramides (Cer) were the most altered lipid molecules in the DR group compared with controls, and as shown in Figure 8B, triglyceride (TG), sphingomyelin (SM), PC, and Cer were the most altered lipid molecules in PDR compared with NPDR.

### 3.5. Combinatorial Biomarker Panel for DR and PDR

The random forest model yielded importance scores and *p*-values, as shown in Supplementary Tables S7 and S8. The panel for the detection of DR consists of 29 indices, and the panel for discriminating PDR from DR patients consists of 22 indices. 

**Figure 9:**
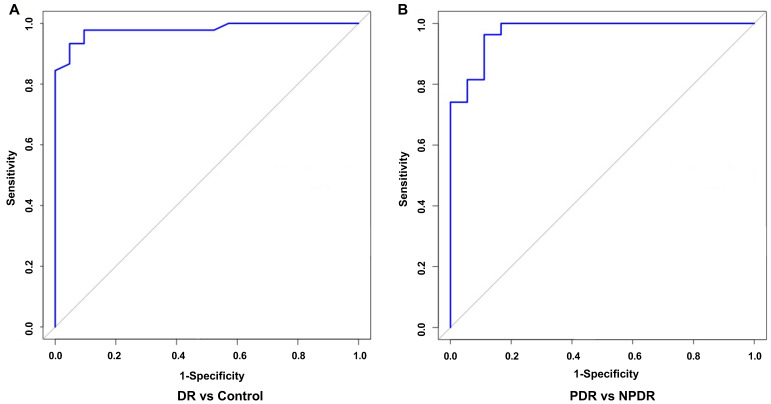
ROC curve of 29 indices in all DR patients and controls (**A**) and 22 indices in PDR patients and NPDR patients (**B**), according to the random forest method. ROC, receiver operating characteristic.

To verify the pertinence of the biomarker panel for DR and PDR, the receiver operating characteristic (ROC) curves were employed. It showed that the area under the curve (AUC) value of the DR biomarker panel was 0.98 (Figure 9A), with sensitivity and specificity of 93% and 95%, respectively. For PDR, AUC was 0.97 (Figure 9B), with sensitivity and specificity of 96% and 89%, respectively. 

## 4. Discussion

The development of omics technologies and bioinformatics have allowed increasing identification of the molecules and pathways involved in DR pathogenesis. He et al. [31] revealed the changed profiles of circRNAs in vitreous humor samples of patients with PDR compared to those of non-diabetes mellitus patients. A recent study demonstrated the altered profiling of exosomal circRNAs in PDR serum and recognized the essential role played by CircFndc3b derived from endothelial cells in pathological angiogenesis [32]. Another study based on proteomics identified 10 dysregulated proteins as potential biomarkers and molecular targets for PDR [33]. 

As a powerful tool for the discovery of important molecules with diagnostic and therapeutic potential, metabolomics analysis has been applied in recent Investig.gations into DR [34]. In addition to plasma, serum, aqueous humor, and vitreous humor samples, fecal samples from DR patients have also been used for metabolomic analysis, combined with high-throughput 16S rRNA analysis, to reveal the profile changes of fecal metabolome and gut microbiome in DR patients [35,36]. Lipidomics analyses have also been used to assess the lipid alterations in DR [27,37,38].

In the present study, we found that multiple metabolites and lipids were significantly changed in the plasma of T2DM patients with proliferative and non-proliferative DR. KEGG analysis was performed based on the significantly altered metabolite levels. As shown in Figure 5, several metabolic pathways were enriched in DR compared with controls and in PDR compared with NPDR. These included amino acid biosynthesis, arginine biosynthesis, protein digestion and absorption, and mineral absorption. In our previous study, we found that many amino acids and derivatives were significantly changed in the retinas of oxygen-induced retinopathy (OIR) model mice [39]. The altered plasma levels of amino acids and derivatives in infants with ROP were also identified by untargeted metabolomics and validated by targeted metabolomics [18,40]. Xiao et al. [41] conducted proteomic profiling of aqueous humor in PDR patients and showed that “protein digestion and absorption” is one of the pathways most enriched by the dysregulated proteins. Interestingly, enriched KEGG pathways were found in the present study and in the previous studies on OIR and ROP, indicating the importance of protein digestion and absorption in DR pathogenesis. 

Our study found an increased level of glutamine and decreased levels of aspartate and glutamate in the plasma of patients with DR (Figure 3). Previous studies have reported that the glutamine-to-glutamate ratio was the best biomarker to distinguish patients with DR [42]. In addition, in the current study, the plasma level of tryptophan was decreased in PDR patients compared with NPDR patients (Figure 4). It has been confirmed that deprivation of tryptophan (and also numerous other amino acids) directly leads to a marked increase in VEGF expression in vitro [43]. Moreover, reducing the catabolism of tryptophan suppresses IL-6/STAT3/VEGF signaling pathway and attenuates angiogenesis in bladder cancer [44]. Therefore, the role and mechanisms played by tryptophan in PDR pathogenesis warrant further Investig.gations.

Several previous studies revealed the metabolite levels in vitreous or aqueous humor samples from patients with DR, which shared some altered metabolites with the current study [45]. For example, proline, glutamine, and citrulline were significantly increased in vitreous samples of PDR [46,47]. In our study, the plasma levels of citrulline and glutamine were up-regulated in the PDR group compared with the NPDR group; meanwhile, that of proline was also increased in patients with DR compared with controls. Asparagine and threonine were increased in the aqueous humor of DR [48]. In the current study, the plasma level of asparagine was also increased in patients with DR. However, that of threonine was decreased in DR patients compared with controls. As intraocular samples indicated, the local changes of metabolites and plasma samples showed the systemic alterations which may be caused by the immune system; the common and specific metabolites, as well as their involved metabolic pathways in DR pathogenesis and development, should be further explored. 

Li et al. [49] found an association between the dietary intake of PCs and the incident risk of T2DM. Dugani et al. [50] indicated that Cers were associated with a risk of prevalence and incidence of T2DM. The present study also showed classification information of the significantly changed lipids (Figure 8). PC and Cer were the main classes of altered lipids in the DR group and PDR subgroup. Therefore, circulation levels of PC and Cer might also be involved in the pathogenesis and development of DR.

Random forest is a bioinformatic technique that allows prediction with high accuracy by using bootstrap aggregation and randomization of predictors [51]. In this study, through random forest analysis, we established potential panels for DR and PDR (Figure 9), which indicated the significant potential for clinical applications of metabolomics and lipidomics. However, further recruitment with a larger number of individuals is needed to confirm the clinical values of each specific metabolite and lipid as diagnostic biomarkers by targeted metabolomics and lipidomics in future studies.

To confirm whether the sex difference is an additional variable to the current study, we applied analysis of covariance according to the significantly altered metabolites and lipids in the DR group (Supplementary Table S9 and Figure S4), and the results showed that no index met the |R| > 0.5 and *p* < 0.05, which confirmed that sex difference may not influence the conclusion.

This study has several limitations. Firstly, the sample size of our study was relatively small. A larger sample size is needed to further analyze the duration of diabetes mellitus and DR, concomitant nephropathy, liver complications, smoking, and other factors. Secondly, further studies of prospective cohort and functional studies are needed to verify whether these differential metabolites and lipids could be therapeutic targets or prognostic indicators of DR treatment. 

## 5. Conclusions

In conclusion, significant alterations of plasma metabolites and lipids were found in T2DM patients with DR. Panels of molecules identified by metabolomics and lipidomics could be potential biomarkers not only for the diagnosis of DR, but also for the distinction between PDR and NPDR cases. Furthermore, these significantly changed metabolites and lipids and their pathways might be involved in DR pathogenesis and may be potential targets for the treatment of patients with DR.

## Figures and Tables

**Table 1:** Clinical characteristics of included subjects of the study.

Characteristics	PDR (*n* = 27)	NPDR (*n* = 18)	Control (*n* = 21)	*p*-Value
Age (years old)	56.59 ± 9.27	55.89 ± 8.82	56.48 ± 5.81	0.9572
Sex (female, %)	13 (48.15%)	11 (61.11%)	9 (42.86%)	0.5080
Diabetic duration (years)	13.00 ± 6.50	11.00 ± 4.75	7.91 ± 5.80	0.0151
BMI (kg/m^2^)	23.18 ± 3.31	24.58 ± 2.40	24.80 ± 1.99	0.0860
Fasting blood glucose (mmol/L)	8.66 ± 3.76	7.72 ± 2.14	6.97 ± 1.60	0.116
HbA1c%	8.04 ± 1.87	8.47 ± 3.10	7.80 ± 2.04	0.2106

## Data Availability

The data presented in this study are available on request from the corresponding author.

## Supplementary Materials

The following supporting information can be downloaded at: https://www.mdpi.com/article/10.3390/pharmaceutics14122751/s1, Supplementary Figure S1: Venn diagrams of significantly changed metabolites with common and uniquely altered metabolites in the three comparison groups under positive (A) and negative ion modes (B); Supplementary Figure S2: Levels of total lipids in DR and control groups (A), and in PDR and NPDR subgroups (B); Supplementary Figure S3: Venn diagrams of significantly changed lipids with common and uniquely altered metabolites in the three comparison groups; Supplementary Figure S4: Heatmap analysis of covariance adjusted for sex; Supplementary Table S1: Significantly altered metabolites between DR (PDR + NPDR) and control groups under ESI positive mode; Supplementary Table S2: Significantly altered metabolites between DR (PDR + NPDR) and control groups under ESI negative mode ; Supplementary Table S3: Significantly altered metabolites between PDR and NPDR subgroups under ESI positive mode; Supplementary Table S4: Significantly altered metabolites between PDR and NPDR subgroups under ESI negative mode; Supplementary Table S5: Significantly altered lipids between DR (PDR + NPDR) and control groups; Supplementary Table S6: Significantly altered lipids between PDR and NPDR subgroups; Supplementary Table S7: The panel for detection of DR consists of 29 indices; Supplementary Table S8: The panel for discriminating PDR from DR patients consists of 22 indices; Supplementary Table S9: The analysis of covariance adjusted for sex.
