# Supplementary material for: Integrated Analysis of Metabolomics and Lipidomics in Plasma of T2DM Patients with Diabetic Retinopathy
Source: Pharmaceutics. 2022 Dec 8;14(12):2751. doi: 10.3390/pharmaceutics14122751 (PMC9786316; doi:10.3390/pharmaceutics14122751)
Supplement: Supplementary file 1 [file pharmaceutics-14-02751-s001.zip › Figure S4.pdf]

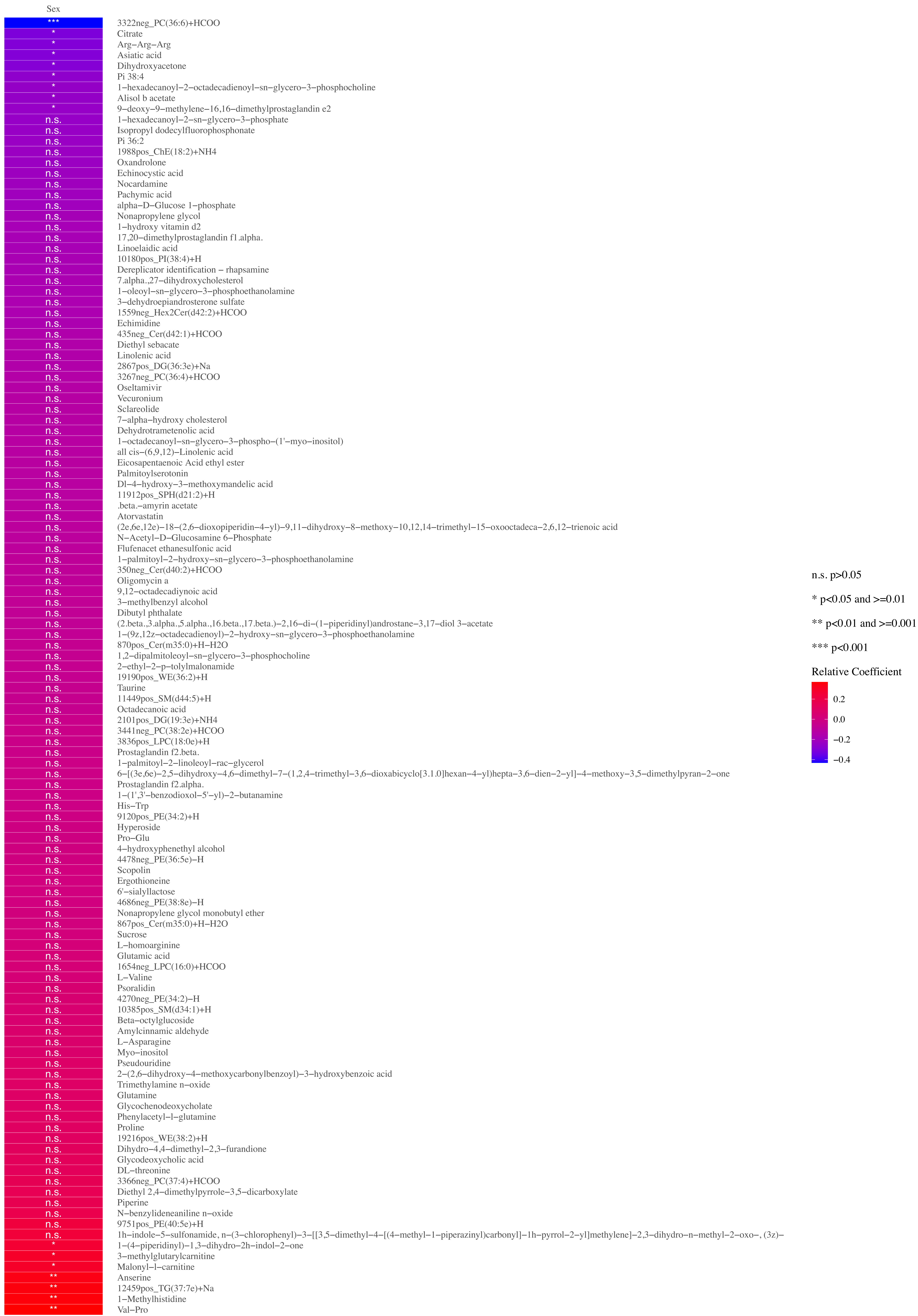
Sex
*** 3322neg_PC(36:6)+HCOO
* Citrate
* Arg−Arg−Arg
* Asiatic acid
* Dihydroxyacetone
* Pi 38:4
* 1−hexadecanoyl−2−octadecadienoyl−sn−glycero−3−phosphocholine
* Alisol b acetate
* 9−deoxy−9−methylene−16,16−dimethylprostaglandin e2
n.s. 1−hexadecanoyl−2−sn−glycero−3−phosphate
n.s. Isopropyl dodecylfluorophosphonate
n.s. Pi 36:2
n.s. 1988pos_ChE(18:2)+NH4
n.s. Oxandrolone
n.s. Echinocystic acid
n.s. Nocardamine
n.s. Pachymic acid
n.s. alpha−D−Glucose 1−phosphate
n.s. Nonapropylene glycol
n.s. 1−hydroxy vitamin d2
n.s. 17,20−dimethylprostaglandin f1.alpha.
n.s. Linoelaidic acid
n.s. 10180pos_PI(38:4)+H
n.s. Dereplicator identification − rhapsamine
n.s. 7.alpha.,27−dihydroxycholesterol
n.s. 1−oleoyl−sn−glycero−3−phosphoethanolamine
n.s. 3−dehydroepiandrosterone sulfate
n.s. 1559neg_Hex2Cer(d42:2)+HCOO
n.s. Echimidine
n.s. 435neg_Cer(d42:1)+HCOO
n.s. Diethyl sebacate
n.s. Linolenic acid
n.s. 2867pos_DG(36:3e)+Na
n.s. 3267neg_PC(36:4)+HCOO
n.s. Oseltamivir
n.s. Vecuronium
n.s. Sclareolide
n.s. 7−alpha−hydroxy cholesterol
n.s. Dehydrotrametenolic acid
n.s. 1−octadecanoyl−sn−glycero−3−phospho−(1'−myo−inositol)
n.s. all cis−(6,9,12)−Linolenic acid
n.s. Eicosapentaenoic Acid ethyl ester
n.s. Palmitoylserotonin
n.s. Dl−4−hydroxy−3−methoxymandelic acid
n.s. 11912pos_SPH(d21:2)+H
n.s. .beta.−amyrin acetate
n.s. Atorvastatin
n.s. (2e,6e,12e)−18−(2,6−dioxopiperidin−4−yl)−9,11−dihydroxy−8−methoxy−10,12,14−trimethyl−15−oxooctadeca−2,6,12−trienoic acid
n.s. N−Acetyl−D−Glucosamine 6−Phosphate
n.s. Flufenacet ethanesulfonic acid
n.s. 1−palmitoyl−2−hydroxy−sn−glycero−3−phosphoethanolamine
n.s. 350neg_Cer(d40:2)+HCOO
n.s. Oligomycin a
n.s. 9,12−octadecadiynoic acid
n.s. 3−methylbenzyl alcohol
n.s. Dibutyl phthalate
n.s. (2.beta.,3.alpha.,5.alpha.,16.beta.,17.beta.)−2,16−di−(1−piperidinyl)androstane−3,17−diol 3−acetate
n.s. 1−(9z,12z−octadecadienoyl)−2−hydroxy−sn−glycero−3−phosphoethanolamine
n.s. 870pos_Cer(m35:0)+H−H2O
n.s. 1,2−dipalmitoleoyl−sn−glycero−3−phosphocholine
n.s. 2−ethyl−2−p−tolylmalonamide
n.s. 19190pos_WE(36:2)+H
n.s. Taurine
n.s. 11449pos_SM(d44:5)+H
n.s. Octadecanoic acid
n.s. 2101pos_DG(19:3e)+NH4
n.s. 3441neg_PC(38:2e)+HCOO
n.s. 3836pos_LPC(18:0e)+H
n.s. Prostaglandin f2.beta.
n.s. 1−palmitoyl−2−linoleoyl−rac−glycerol
n.s. 6−[(3e,6e)−2,5−dihydroxy−4,6−dimethyl−7−(1,2,4−trimethyl−3,6−dioxabicyclo[3.1.0]hexan−4−yl)hepta−3,6−dien−2−yl]−4−methoxy−3,5−dimethylpyran−2−one
n.s. Prostaglandin f2.alpha.
n.s. 1−(1',3'−benzodioxol−5'−yl)−2−butanamine
n.s. His−Trp
n.s. 9120pos_PE(34:2)+H
n.s. Hyperoside
n.s. Pro−Glu
n.s. 4−hydroxyphenethyl alcohol
n.s. 4478neg_PE(36:5e)−H
n.s. Scopolin
n.s. Ergothioneine
n.s. 6'−sialyllactose
n.s. 4686neg_PE(38:8e)−H
n.s. Nonapropylene glycol monobutyl ether
n.s. 867pos_Cer(m35:0)+H−H2O
n.s. Sucrose
n.s. L−homoarginine
n.s. Glutamic acid
n.s. 1654neg_LPC(16:0)+HCOO
n.s. L−Valine
n.s. Psoralidin
n.s. 4270neg_PE(34:2)−H
n.s. 10385pos_SM(d34:1)+H
n.s. Beta−octylglucoside
n.s. Amylcinnamic aldehyde
n.s. L−Asparagine
n.s. Myo−inositol
n.s. Pseudouridine
n.s. 2−(2,6−dihydroxy−4−methoxycarbonylbenzoyl)−3−hydroxybenzoic acid
n.s. Trimethylamine n−oxide
n.s. Glutamine
n.s. Glycochenodeoxycholate
n.s. Phenylacetyl−l−glutamine
n.s. Proline
n.s. 19216pos_WE(38:2)+H
n.s. Dihydro−4,4−dimethyl−2,3−furandione
n.s. Glycodeoxycholic acid
n.s. DL−threonine
n.s. 3366neg_PC(37:4)+HCOO
n.s. Diethyl 2,4−dimethylpyrrole−3,5−dicarboxylate
n.s. Piperine
n.s. N−benzylideneaniline n−oxide
n.s. 9751pos_PE(40:5e)+H
n.s. 1h−indole−5−sulfonamide, n−(3−chlorophenyl)−3−[[3,5−dimethyl−4−[(4−methyl−1−piperazinyl)carbonyl]−1h−pyrrol−2−yl]methylene]−2,3−dihydro−n−methyl−2−oxo−, (3z)−
* 1−(4−piperidinyl)−1,3−dihydro−2h−indol−2−one
* 3−methylglutarylcarnitine
* Malonyl−l−carnitine
** Anserine
** 12459pos_TG(37:7e)+Na
** 1−Methylhistidine
** Val−Pro
n.s. p>0.05
* p<0.05 and >=0.01
** p<0.01 and >=0.001
*** p<0.001
Relative Coefficient
0.2
0.0
−0.2
−0.4
