# Supplementary figures and images for: Integrated Analysis of Metabolomics and Lipidomics in Plasma of T2DM Patients with Diabetic Retinopathy
Source: Pharmaceutics. 2022 Dec 8;14(12):2751. doi: 10.3390/pharmaceutics14122751 (PMC9786316; doi:10.3390/pharmaceutics14122751)

**A**

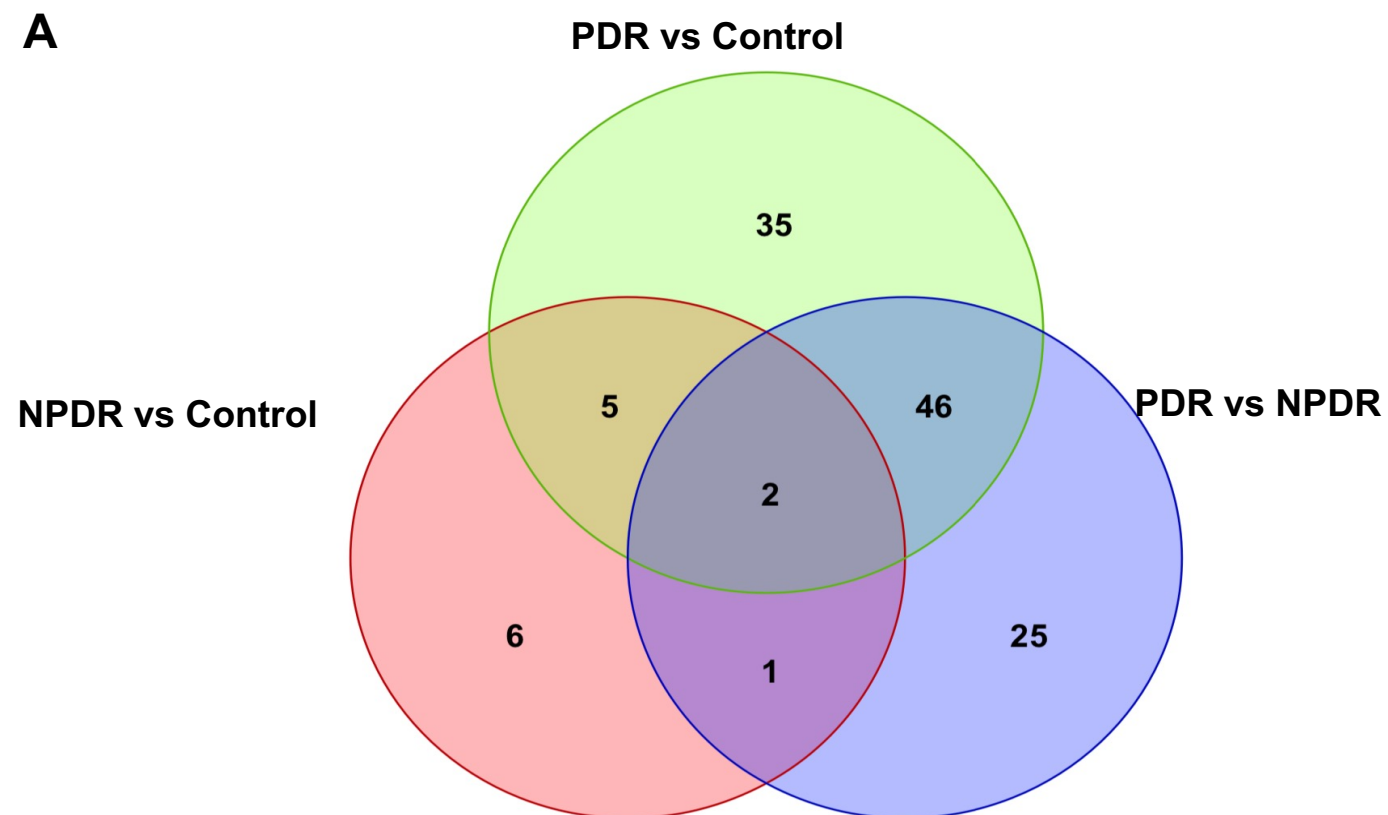

**B**

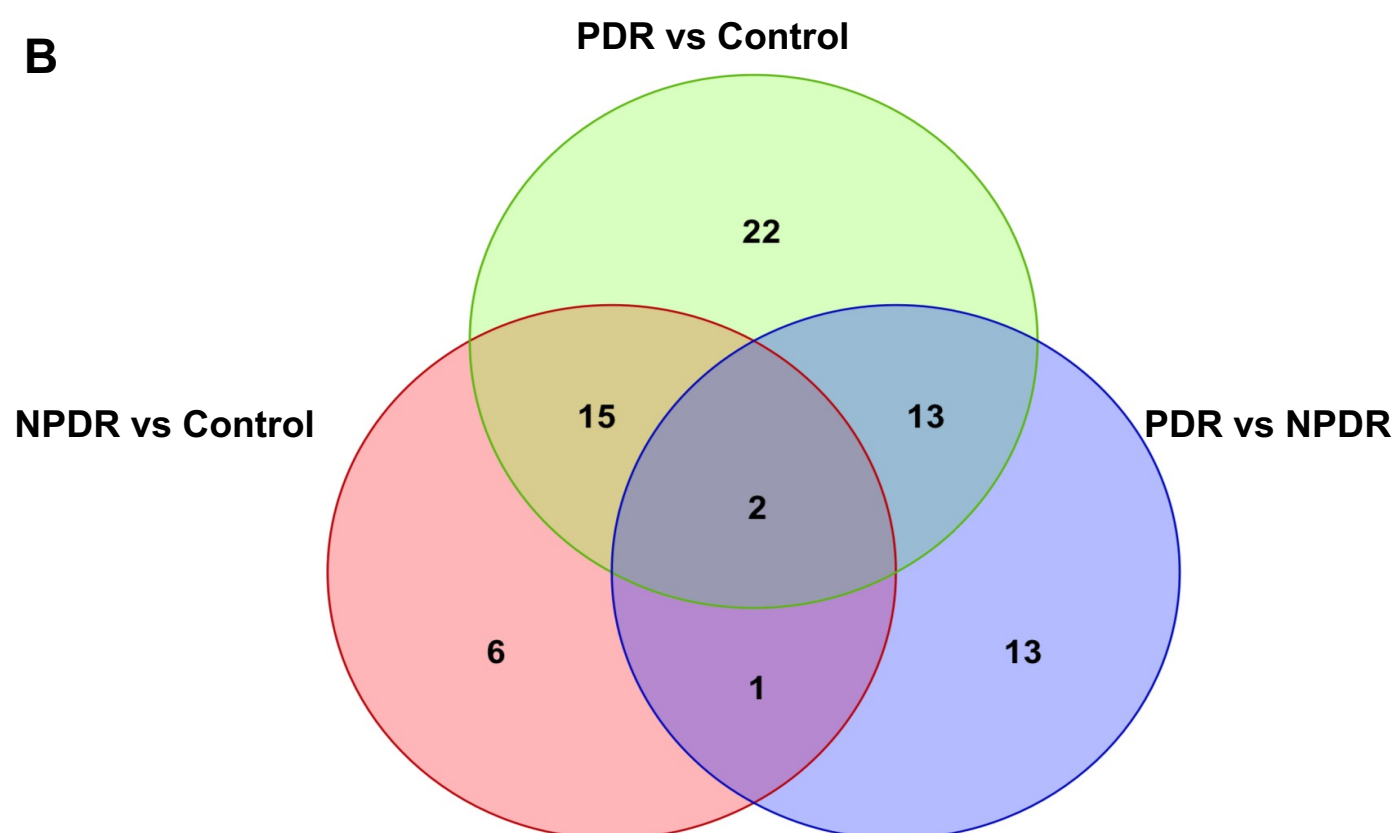

Supplement: Supplementary file 1 [file pharmaceutics-14-02751-s001.zip › Figure S1.pdf]

**A**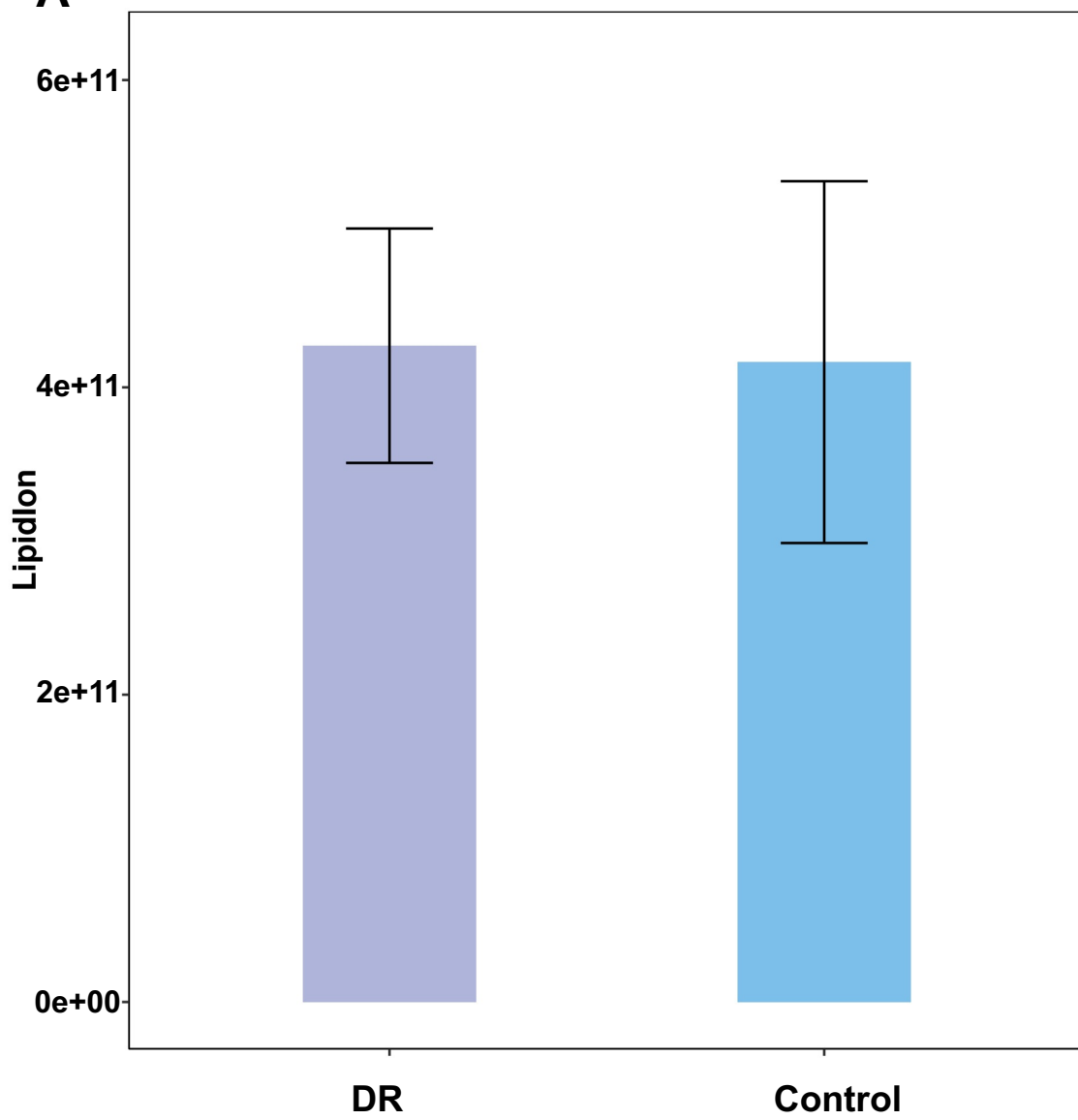**B**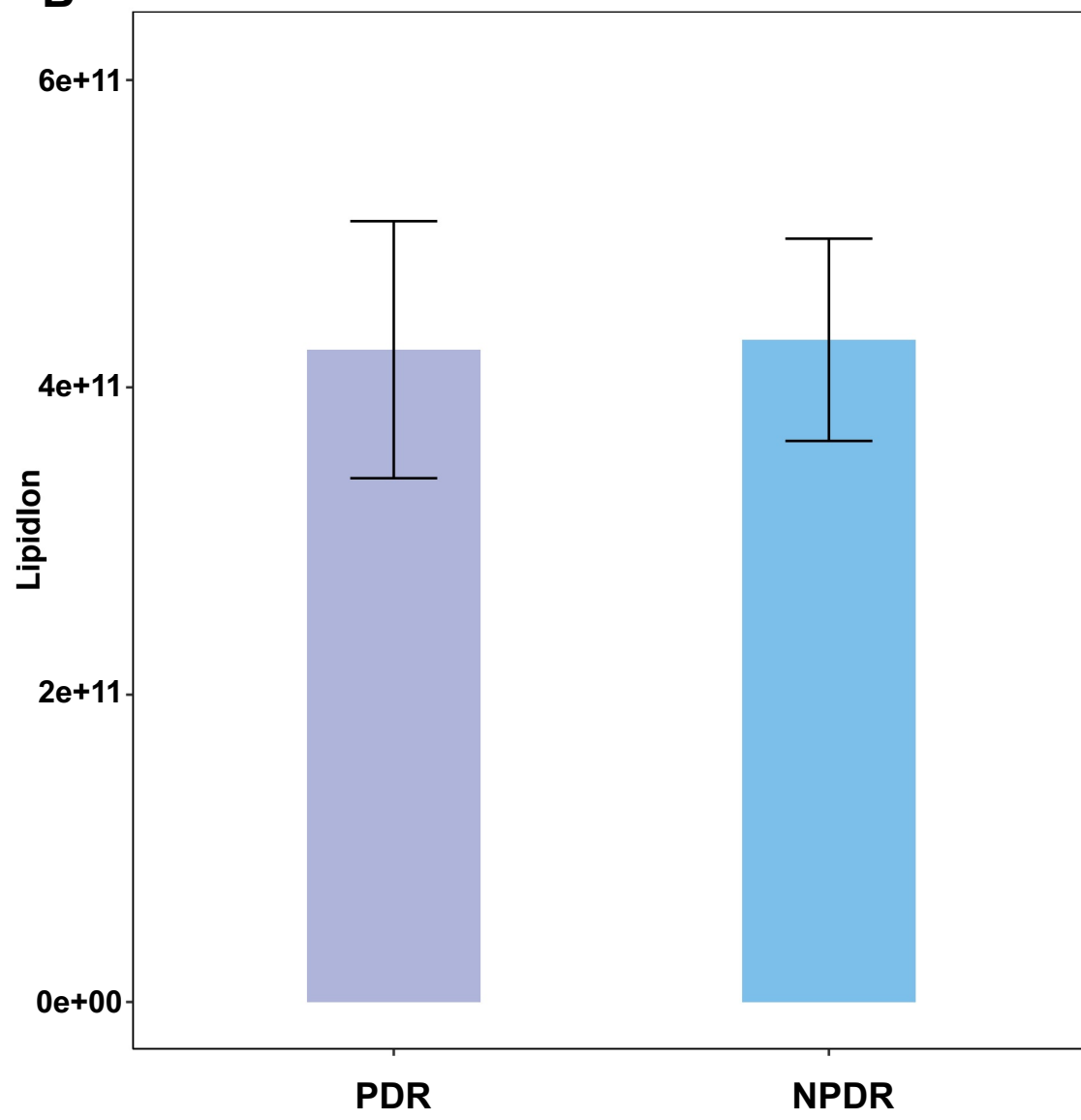

Supplement: Supplementary file 1 [file pharmaceutics-14-02751-s001.zip › Figure S2.pdf]

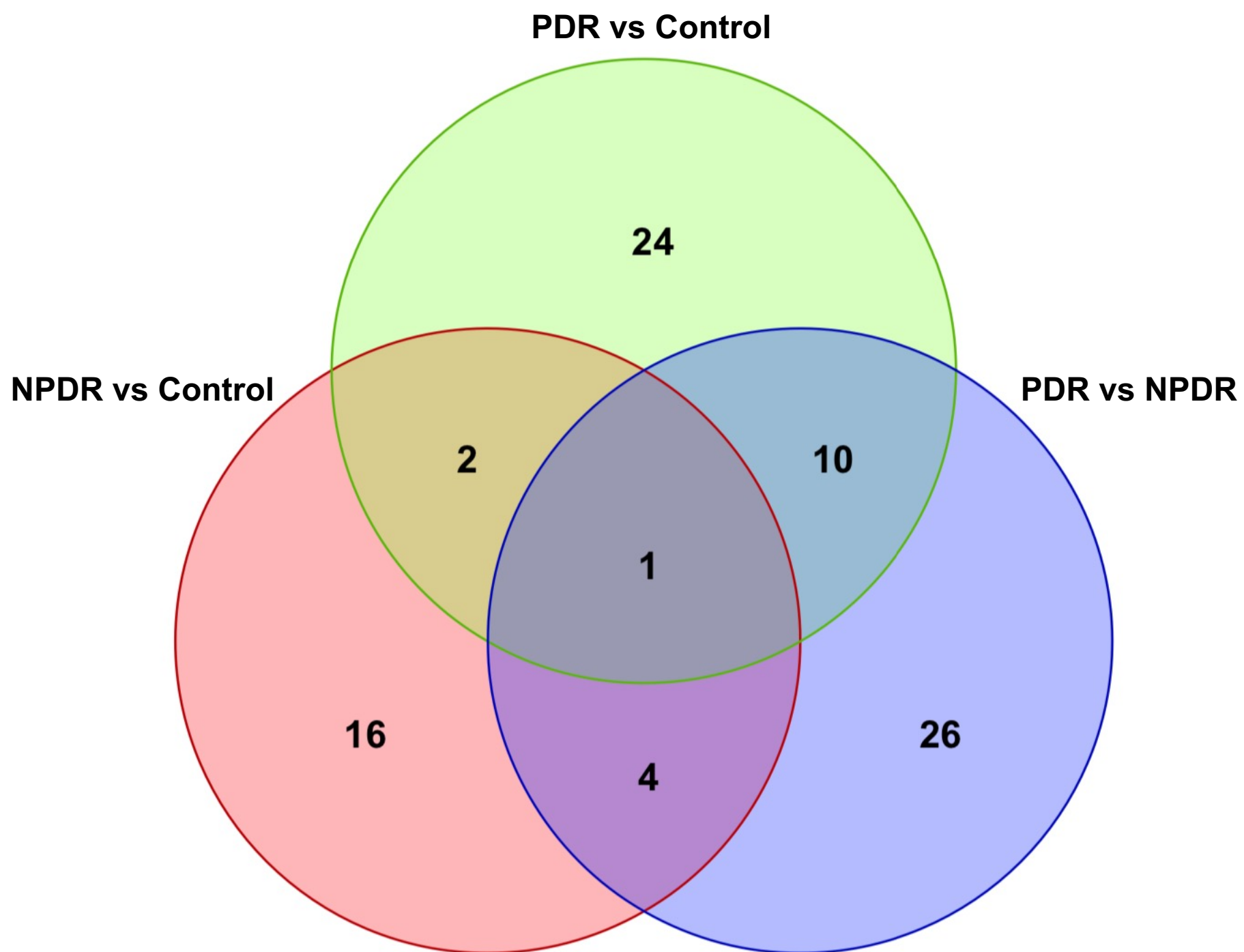

Supplement: Supplementary file 1 [file pharmaceutics-14-02751-s001.zip › Figure S3.pdf]
